# Radicalisation in adolescents: mental health considerations for violent extremism

**DOI:** 10.1177/10398562241292209

**Published:** 2024-10-14

**Authors:** John Kasinathan, Annie Parsons

**Affiliations:** Psychiatry and Mental Health, 98994 University of New South Wales Medicine & Health, Sydney, NSW, Australia; Adolescent Forensic Mental Health, 161777 Justice Health and Forensic Mental Health Network, Matraville, NSW, Australia; Adolescent Forensic Mental Health, 161777 Justice Health and Forensic Mental Health Network, Matraville, NSW, Australia

**Keywords:** radicalisation, violent extremism, adolescents, youth, treatment

## Abstract

**Objective:**

To outline current understanding and recommended mental health and psychiatric considerations for radicalisation and violent extremism among adolescents.

**Method:**

Overview of recent research regarding violent extremism in adolescents and relationships with mental illness and other psychosocial determinants. Relevant international and Australasian research is outlined, with an emphasis on adolescents. Psychiatric considerations, intervention and policy implications will be explored.

**Results:**

Adolescents who become radicalised form a heterogenous group with complex, multifaceted needs from mental disorder, familial, societal and/or environmental contributions. Thus, assessment and management need to be individualised. Mental health clinicians working with at-risk and radicalised adolescents should maintain a high index of suspicion for mental illness (particularly psychosis and depressive disorder) and neurodevelopmental disorder. Identified psychiatric conditions warrant prioritised mental health treatment. There may be a relationship between specific psychopathology and certain ideological beliefs and behaviours.

**Conclusions:**

Radicalised adolescents pose challenges with risk of serious harm to others, presentation complexity, multifactorial contributors and associations with varied psychopathology. All adolescents at risk of radicalisation or who are radicalised, should receive comprehensive mental health assessment and prompt assertive treatment of identified psychiatric conditions.

Youth radicalisation is of rising concern globally^1^ and in Australia and New Zealand, with jurisdictions implementing rehabilitation programs targeting youth who have committed terrorist offences and those at risk of radicalisation.^2^ Studies regarding radicalised youth frequently include data from persons aged 12 to 25. Where possible this paper will focus on those 18 years and younger.

**Ethical statement:** There are no human participants in this article and informed consent was not required.

## Recent Australian events involving adolescents

Recent events arrested media attention with the arrest and detention of several Sydney teenagers on terrorism-related charges following the alleged stabbing of an Assyrian Orthodox bishop Mar Mari Emmanuel Bishop by a 16-year-old male at Christ the Good Shepherd Church in Wakeley New South Wales (NSW) on 15 April 2024.^3^ The boy was remanded into youth custody after allegedly stabbing the bishop during a live-streamed service. The conservative bishop’s reported comments about Islam and the Prophet Muhammad had been widely shared online. Three days later NSW Police released a statement saying that the boy was interviewed by Joint Counter Terrorism officers and charged with committing a terrorist act.^3^

ABC’s 7.30 report aired an interview with the accused’s parents who described their boy’s long history of anger difficulties, and childhood behaviours such as toe walking, repetitive movements and inattention suggestive of autism and ADHD.^4^ They described how he saw a succession of counsellors for aggression and behavioural issues starting in primary school (see Table 1 for excerpts from the ABC transcript).

**Table 1.** Excerpts from ABC 7.30 report transcript (posted 29 April 2024)^4^

After the boy left school last year, a second psychologist urged his parents to seek a diagnosis of autism. But by their account, life and death got in the way.
There was an urgent trip to Lebanon to support a dying uncle and the boy’s mother was seriously unwell for months. She said her son was increasingly unruly and defiant.
He refused to go to TAFE and couldn’t find a job. At home, he spent long nights in the dark in his room on his phone. His parents insisted they had no idea what he was doing.
ABC Investigations has discovered that in the months leading up to the stabbing, the boy was interacting with extremists in Australia and following extremist accounts online. He liked a series of violent extremist posts published on Instagram, including a video of Osama bin Laden encouraging followers to die for the cause.
According to court documents, the assessment cleared the boy of a mental health disorder by the following day, but his family says he now needs a full psychiatric evaluation.
‘If he’s truly religious, like we raised him, he wouldn’t have done that’, his mother said. ‘He’s made a big mistake, a very big mistake’. ‘But I believe he wasn’t in his right mind’.

A week after the Wakeley incident, seven adolescents were arrested and a further five questioned by police after raids in south-west Sydney by the joint counterterrorism team, who reported a ‘network’ of adolescents who shared ‘similar violent extremist ideology’.^5^ Five teenagers were subsequently reported as facing charges following raids in Sydney and Goulburn. Two 16-year-old males were charged with ‘conspiring to engage in any act in preparation for, or planning, a terrorist act’. One 17-year-old boy was charged with ‘conspiring to engage in an act in preparation for, or planning, a terrorist act and custody of a knife in a public place’. Two boys aged 14 and 17 were charged with possessing or controlling extremist material, including images of beheading and instruction videos on how to make a bomb.^5^

Shortly after, on 5 May 2024 in Perth, police reportedly shot dead a 16-year-old boy who allegedly stabbed another man with a kitchen knife, before running at police.^6^ Police said the teenager was ‘radicalised’ online. He reportedly was part of a countering violent extremism intervention program for 2 years, which aimed to identify individuals at risk of radicalising and provide tailored services to reduce that risk.^6^

## Definitions

Definitions of relevant terms are apposite at this point, see Table 2.^7–11^ In the literature, radicalisation generally constitutes beliefs, whereas violent extremism is the behavioural outcome of those beliefs.^12^ Violent extremism can be delineated as religious, the growing far-right movement, far-left and single-issue extremism. Notably, most existing research on religious extremism refers to Islamic extremist ideology such as Islamic State.^1^ Far-right ideologies, such as white-nationalism, justify violence based on belief of superiority to other cultures and races.^7^ Far-left ideologies advocate for social egalitarianism but are hostile to procedural and behavioural norms of liberal democracy; for example, Antifa. Single-issue extremism may canvas specific ideologies such as animal liberation or anti-gun control.^9^

**Table 2.** Definitions

***Terrorism:*** The unlawful use of violence or threat of violence, often motivated by religious, political, or other ideological beliefs, to instill fear and coerce governments or societies in pursuit of goals that are usually political.^10^
***Ideology:*** A conscious and unconscious set of ideas … that imposes a pattern, structure, and interpretation on how we read facts, events, occurrences and actions.^7^
***Radicalisation:*** The psychological, emotional, and behavioural process by which an individual or group adopts an ideology that promotes the use of violence for the attainment of political, economic, religious, or social goals.^8^
***Violent extremism:*** An extension of radicalisation from a relatively benign expression of a viewpoint to the use of violence to achieve a particular goal.^9^
***Countering violent extremism (CVE):*** Efforts designed to reduce the risk of home-grown terrorism by strengthening Australia’s resilience to radicalisation and assisting individuals to disengage from violent extremist influence and beliefs.^7^

## Why consider adolescents differently to adults

While violent extremism is a potentially high-impact phenomenon, violent extremist acts are a fortunately a low-incidence problem in Australia. ASIO estimated that less than 0.1% of the Australian population will have any association with a violent extremist group.^12^ At the same time, an Australian Government commissioned report described the risk of Australians under 18 being radicalised to engage in terrorist acts as ‘significant and likely to continue’.^12^ Globally, a main predictor of engagement in violent extremism is young age, with those under 30 years making up the bulk of violent radical organisations.^1^ Islamic State has been described as particularly adept at recruiting youth, appealing to a sense of camaraderie and belonging. And music, clothes and social media have been used by far-right and white supremacist groups to target teenagers.^2^

Extremists acts by adolescents in Australia prior to 2024 include 15-year-old Farhad Khalil Jabar who shot dead a NSW police employee, 18-year-old Numan Haider who was shot by police in Melbourne when he attempted to stab them, two 17-year-olds Taha El Baf and Abdullah Elmir who joined Islamic State in Syria, and an unnamed 16-year-old in NSW sentenced to 16 years for plotting a terrorist act.^1^

Born from these concerns, countering violent extremism (CVE) programs were developed across Australia and New Zealand, with secondary prevention efforts for individuals identified at risk of radicalisation.^2^ Multi-agency responses are emphasised to address multiple needs contributing to risk, including mental health needs. Therefore, mainstream child and adolescent mental health services, and certainly adolescent forensic mental health clinicians may regularly work with radicalised adolescents and/or involved in CVE initiatives. Familiarity with the evidence base and CVE principles is important to avoid siloing and stigmatisation while working towards better outcomes for adolescents and the community.

While we initially provide an overview of relevant adult literature, children are not miniature adults. Adolescence presents unique developmental tasks and challenges, such as the search for identity, reduced capability for emotion regulation, and increased need for social connection.^13^ These aspects may increase vulnerability to radicalisation. Adolescence is also a period of psychiatric diagnostic uncertainty; serious mental illness (such as psychotic and major mood disorders) often emerges between the ages of 12 and 25.^14^ Further, adolescents in custody have much higher rates of psychotic disorder, mood disorders, neurodevelopmental disorders, trauma, educational disengagement and social marginalisation than their age-matched peers.^15^ Might this also be true for radicalised adolescents?

## Adult psychopathology and violent extremism

In the adult literature, it was historically purported that little to no mental disorder existed in terrorist samples, that the terrorist population was ‘mentally healthier’ than the general population. However, this has been largely debunked, with acknowledgement of marked heterogeneity and varied rates of psychopathology across a broad range of terrorist activities.^16^

While no single terrorist profile exists, there seem to be different types of terrorist offending, the most pronounced distinction: lone actors versus those part of a group.^16^ These typologies appear distinct in terms of drivers and criminogenic needs. One study found lone-actor terrorists had rates of schizophrenia at 8%, delusional disorder 2% and autism spectrum disorder 2.5%, which were all higher than rates of those diagnoses in either group actors or the general population.^16^

A subsequent systematic review of 25 studies across 28 samples of radicalised adults, reported highly heterogenous prevalence rates of mental health problems ranging from zero to 57%.^17^ Variability was attributed to differing definitions and heterogeneity of the aggregated sample. When results were pooled from studies where sample sizes were known (*n* = 1705 subjects), the rate of confirmed mental illness diagnosis was 14.4%, however no one diagnosis stood out. Where studies had privileged access to police or judicial data (*n* = 283), mental illness diagnoses occurred at 16.96%. In studies purely based upon open sources (*n* = 1089), diagnoses were present 9.82%.^17^ The authors highlighted that rarely were mental health problems the sole issue, sometimes they compounded other problems and vice versa. Constellations of risk and protective factors likely differ between people who may have similar mental health problems; thus, CVE efforts need to be tailored to all identified risk needs and sensitive to relevant mental health concerns rather than simply their presence or absence.^17^

## Adolescent psychopathology and violent extremism

The adolescent literature is an emerging and studies are few.^1^ The following studies within the last 5 years explore relationships between mental disorder and adolescent radicalisation.

Campelo et al.^18^ reported on 20 adolescents assessed in a French child psychiatry department radicalisation prevention unit. Data was drawn from psychiatry consultations, family and individual therapy, and psychological testing. The sample was aged between 14 and 20 years, four females and 16 males. Four young people (20%) were diagnosed with psychotic disorder. For these four, radical proselytism (efforts to convert to radical beliefs) was directly related to intensity of their psychotic symptoms, and both reduced gradually following antipsychotic treatment. None of the four were convicted for ‘criminal association to commit terrorism’; the French dispensation was akin to ‘act proven not criminally responsible’ in Australian legislation.

Other adolescents in the sample displayed a combination of various psychiatric conditions and problematic behaviours. Eleven presented with conduct disorder, 10 had social anxiety disorder with social isolation before appearance of radical conduct, five had substance use, five had engaged in risky sexual behaviours, three had suicidal ideas and/or attempts, and three had bulimia nervosa. Of the 11 adolescents with conduct disorders, four showed a transgression against parental rules (e.g. running away); four committed other prior aggressions; and three had combined histories of rule transgression, aggression, and robbery.

The non-psychotic youth shared elevated rates of other stressors including intrafamilial violence, sexual abuse, imprisonment of family members, traumatic family histories, and significant psychological control or dependence phenomena. Their profile was distinct from the psychotic group, none of whom had been in contact with a member of a terrorist organisation. Those with psychosis often had converted to Islam and lived in single-mother families with an absent father and experienced less conflict in the family environment than non-psychotic subjects. Motivational aspects linked to interest in violence and weapons, included adventure, fighting, ‘male values’, lack of self-esteem, and a lack of interest in searching for tenderness, which correlated significantly with a worse outcome.^18^

## Adolescent psychopathology, ideology and grievances

Duits et al. examined differences between adult (age 22-60) and juvenile (age 15-21) terrorist offenders with regards to psychopathology, ideology and grievances.^19^ Trajectory models conceptualise radicalisation as progression through stages. The role of perceived injustices and grievances in radicalisation progression has been theorised, that these feelings are triggered through events ranging from loss of a family member, experiencing discrimination and online content of people suffering who remind them of family.

Investigators drew from coded data from the European database of convicted terrorist offenders, forensic mental health reports from police, public prosecution, court files, Ministry of Justice and prison administration, and probation reports, from judicial institutions in the Netherlands, Belgium, six German federal states, Austria, and Sweden. Clinical symptoms of mental disorder (rather than diagnoses of mental disorder, so a lower threshold for caseness) were found in 81% of the young terrorist offenders and 73% of the adult terrorist offenders. In contrast to the adult study mentioned above,^16^ authors did not find a significant difference between lone-actor or group offenders.^19^

Cluster B personality disorder/traits (32%), depressive symptoms (23%), intellectual disability (23%), psychosis (16%), and substance use disorder (13%) were most frequently reported.^19^ Psychopathology co-occurrence was reported in 65% of the 31 young terrorist offenders; similar to multimorbidity observed in the general youth offender population.

A positive and moderate association (*p* = .033) between depressive symptoms and grievances about perceived injustices was found only in the juvenile group. In both young and adult terrorist offenders, grievances about perceived injustice were related to relationship problems. More than 75% of both young and adult terrorist offenders who were diagnosed with a mental disorder also met criteria for clinically relevant personality traits, such as poor regulation of aggression, feelings of anger, distrust, paranoid feelings or relationship problems.^19^

## Australian data regarding adolescent radicalisation

Cherney et al.’s analysis of 33 cases of Australian adolescents aged 19 and younger, drew from a database with open sources, court documents and media reports to compile variables on radicalised individuals.^1^ Radicalisation was associated with poor educational achievement, mental health problems, and active social media engagement (including dissemination of content, appearing in propaganda videos, or direct communication with extremists), rather than passive engagement such as viewing images and videos but not interacting. There were associations with radicalised network exposure, personal grievances, such as feeling attached to a group believed to be oppressed, and triggering events. For the latter, a significant event appeared to precipitate or accelerate radicalisation in 88% of cases. Events included circumstances such as a humiliating police search, passport cancellation, relationship breakdown, and family bereavement.

Commitment to ideology that justifies the use of violence, grievances about perceived injustice, and the anger or outrage in response to perceived injustice are specific for violent extremism. These factors form important indicators in structured professional risk assessment tools, such as the Violent Extremism Risk Assessment (VERA-2R)^20^ and Terrorist Radicalization Assessment Protocol (TRAP-18).^21^

Of the Australian sample,^1^ 42% had a history of mental illness or personality disorder, relatively high compared to other studies’ rates ranging from 8 to 31%. Diagnosis was usually made before the individual engaged in terrorism activities. 24% had history of drug or alcohol use and 18% had a prior juvenile record for violent and non-violent crime. Interestingly, most of the sample had close family relationships which are often thought of as a prosocial buffer to deviant pathways. The authors hypothesised that individuals in this sample were exposed to religious beliefs amongst family members who may have exacerbated, reinforced, or at least failed to challenge, emerging extremist ideology.^1^

## Childhood adversity and trauma

Childhood trauma has a cumulative and long-term impact on later-life health and wellbeing.^22^ Childhood trauma can lead to a range of symptoms commonly seen in youth in contact with the criminal justice system, including emotional and behavioural dysregulation, attention and cognitive deficits and executive dysfunction.^23^ Childhood maltreatment is a robust predictor of later adolescent aggression.^24^ Adverse childhood experiences (ACEs) include direct experiences of abuse and or neglect, and more indirect trauma such as exposure to family violence, mental illness and substance use.^25^

Almost two-thirds (63%) of a sample of US-based former white supremacists experienced four or more ACEs before age 18, comparable to 55% of a young offender group, and much higher than 16% of a general population group.^26^ Thus ACEs correlate with both ideological and non-ideological offending, raising the question whether joining an extremist group is primarily ideologically driven or rather may reflect other drivers for sense of belonging and stability.^26^

## Gender considerations

Male gender and associated values appear related to adolescent extremism although some female adolescents are involved. For example, Campelo et al’s 20 adolescents in a French child psychiatry department radicalisation prevention program had four females.^18^ Cherney et al.’s analysis of 33 Australian adolescents had 9.1% of the sample (*n* = 3) female. Cherney et al.^1^ noted that despite their sample’s age, just over one-third were married (all Muslim) and within this context, female partners appeared to play some role in the radicalisation process. Cherney et al.^1^ further examined case details of the three females in their sample: all had romantic relations with other radicalised individuals and, in some cases, openly encouraged extremist acts of their male partners. The authors inferred that for some young people, marriage and bonding between partners is part of the radicalisation process.^1^

## CVE intervention approaches for adolescents

Some jurisdictions such as NSW offer voluntary case management for adolescents vulnerable to violent extremism, aiming to build protective factors, a positive sense of identity, belonging and self-worth.^2^ CVE interventions can span from individual psychological work to recreational activities, mentoring and vocational programs.

A recent Australian review of rehabilitation for radicalised adolescents, recommended several core features to underpin interventions including:^27^

a) Addressing multiple needs that on their own might not lead to violent extremism but when coupled with other social and cognitive deficits, can compound risk.
b) Ensuring family participation in interventions.
c) Generating change through cognitive behavioural therapy, and assistance with emotional regulation, moral reasoning, empathy and cognitive development.
d) Ensuring interventions are trauma-informed, non-stigmatising and non-judgemental (noting stigmatising interventions can further alienate youths, contributing to grievances).
e) Informal engagement involving non-clinical and non-vocational/educational activities are essential.
f) Ensuring programs are developmentally appropriate; expectations about change must consider developmental and emotional capacities.
g) Involved professionals are thoroughly trained with up-to-date knowledge regarding drivers of violent extremism and online influences.
h) Multi-agency responses are vital, whilst not duplicating supports, ensuring interventions are complimentary and add value.

## Implications for mental health clinicians and services

Relationships between mental disorders and radicalisation to violent extremism are evident in a significant proportion, though causality is not clear-cut. Radicalised adolescents form a heterogenous group with complex needs, including higher rates of mental disorders. Risk factors involve not only mental disorder, but also issues relating to relationships, discrimination, unemployment, life changes, traumatic experiences, and substance use, all of which need to be considered in risk appraisal. Likewise, risk management must be individualised and tailored to identified needs.

For mental health clinicians working with radicalised adolescents, the available evidence supports maintaining high indices of suspicion for mental illness (in particular, psychosis and depressive disorder) and neurodevelopmental disorder, ensuring that mental health treatment is prioritised when conditions are identified. Where mental illness is present, psychiatric treatment may have direct amelioration upon ideological beliefs and behaviours. However, there is no single psychiatric profile for this population. Clinicians should avoid mad / bad dichotomies and embrace the evident complexity with included mental health contributors.

Structured professional risk assessment can inform mental health treatment approaches to target modifiable factors that drive violence and aggression in adolescents. Relevant tools include the Structured Assessment of Violence Risk in Youth (SAVRY),^24^ Violent Extremism Risk Assessment (VERA-2R)^20^ and Terrorist Radicalization Assessment Protocol (TRAP-18).^21^ Adolescent radicalisation may involve anger towards Australian or New Zealand society and rejection of values,^1^ thus it is possible that mental health clinicians may be viewed as part of a ‘repressive state’. Potential risk of harm to treatment providers needs to be managed thoughtfully and sensibly, akin to a delusional patient who may incorporate treating clinicians into their false beliefs. Therapeutic relationship building is a key protective factor. Treatment of specific mental disorders may further reduce risks. Provided usual precautions are followed, like any patient with or without forensic issues, risk to treating clinicians is in our view manageable.

Finally, outcomes between mental disorder and adolescent radicalisation appear to be mixed. On one hand, Campelo et al. reported four young people diagnosed with psychotic disorders, their radical proselytism was directly related to psychotic symptom intensity, and both reduced gradually following antipsychotic treatment.^18^ On the other hand, ABC’s 7.30 report aired an interview with parents of an adolescent male charged with a violent extremist offence; they described their son’s extensive anger difficulties, childhood toe walking, repetitive movements and inattention suggestive of autism and ADHD.^4^ He saw successive counsellors and this did not prevent radicalisation. Further quality studies will offer greater clarity than media case reports.

Adolescent radicalisation arises from a multimodal landscape, with mental disorder(s), developmental immaturity, personality factors, familial influences, societal factors and/or environmental contributors all potentially playing a role. Thus, interagency communication, trauma-informed approaches, developmentally appropriate interventions and timely clinically indicated mental health treatment, are all key to support the needs of radicalised adolescents and, as a result, safer communities.

## Data Availability

Data sharing not applicable to this article as no datasets were generated or analysed during the current study.*
